# Supplementary material for: The brain hierarchically represents the past and future during multistep anticipation
Source: Nat Commun. 2024 Oct 22;15:9094. doi: 10.1038/s41467-024-53293-3 (PMC11496687; doi:10.1038/s41467-024-53293-3)
Supplement: Supplementary file 3 — Reporting Summary [file 41467_2024_53293_MOESM3_ESM.pdf]

nature portfolio

Corresponding author(s): Hannah Tarder-Stoll

Last updated by author(s): September 6, 2024

# Reporting Summary

Nature Portfolio wishes to improve the reproducibility of the work that we publish. This form provides structure for consistency and transparency in reporting. For further information on Nature Portfolio policies, see our Editorial Policies and the Editorial Policy Checklist.

## Statistics

For all statistical analyses, confirm that the following items are present in the figure legend, table legend, main text, or Methods section.

| n/a | Confirmed | |
|---|---|---|
| ☐ | ☒ | The exact sample size (*n*) for each experimental group/condition, given as a discrete number and unit of measurement |
| ☐ | ☒ | A statement on whether measurements were taken from distinct samples or whether the same sample was measured repeatedly |
| ☐ | ☒ | The statistical test(s) used AND whether they are one- or two-sided<br>*Only common tests should be described solely by name; describe more complex techniques in the Methods section.* |
| ☐ | ☒ | A description of all covariates tested |
| ☐ | ☒ | A description of any assumptions or corrections, such as tests of normality and adjustment for multiple comparisons |
| ☐ | ☒ | A full description of the statistical parameters including central tendency (e.g. means) or other basic estimates (e.g. regression coefficient) AND variation (e.g. standard deviation) or associated estimates of uncertainty (e.g. confidence intervals) |
| ☐ | ☒ | For null hypothesis testing, the test statistic (e.g. *F*, *t*, *r*) with confidence intervals, effect sizes, degrees of freedom and *P* value noted<br>*Give P values as exact values whenever suitable.* |
| ☒ | ☐ | For Bayesian analysis, information on the choice of priors and Markov chain Monte Carlo settings |
| ☐ | ☒ | For hierarchical and complex designs, identification of the appropriate level for tests and full reporting of outcomes |
| ☐ | ☒ | Estimates of effect sizes (e.g. Cohen's *d*, Pearson's *r*), indicating how they were calculated |

*Our web collection on statistics for biologists contains articles on many of the points above.*

## Software and code

Policy information about availability of computer code

| | |
|---|---|
| Data collection | Data was collected using PsychoPy version 2. |
| Data analysis | After preprocessing, all fMRI analyses were performed in Python and R. Pattern similarity analyses were performed using custom code in Python 3. Statistical analysis comparing pattern similarity values across conditions, correlations between fMRI results and behavior, and visualizations were performed using custom code in R version 4.4.0 with the help of the lme4 package, version 1.1.35.3. Code to reproduce all figures and statistical analyses in the manuscript and supplement is available at https://github.com/hannahtarder-stoll/predNav. |

For manuscripts utilizing custom algorithms or software that are central to the research but not yet described in published literature, software must be made available to editors and reviewers. We strongly encourage code deposition in a community repository (e.g. GitHub). See the Nature Portfolio guidelines for submitting code & software for further information.

## Data

Policy information about availability of data

All manuscripts must include a data availability statement. This statement should provide the following information, where applicable:

- Accession codes, unique identifiers, or web links for publicly available datasets
- A description of any restrictions on data availability
- For clinical datasets or third party data, please ensure that the statement adheres to our policy

Source data is provided with this paper. fMRI data is publicly available and can be accessed at https://openneuro.org/datasets/ds005125.

# Research involving human participants, their data, or biological material

Policy information about studies with human participants or human data. See also policy information about sex, gender (identity/presentation), and sexual orientation and race, ethnicity and racism.

| | |
|---|---|
| Reporting on sex and gender | The findings apply to all genders. We have indicated that sex and/or gender was based on self-report and all genders were included in the study. The study was not designed to test gender differences and we had no reasons to expect there to be any based on the existing literature. We have included self reported gender for each participant in the source data. |
| Reporting on race, ethnicity, or other socially relevant groupings | Demographics questionnaires revealed that we had 19 participants who identified as White, 12 participants who identified as Asian, and 1 participant who identified as Black based on self report. We did not control for race or ethnicity in our analyses as the study was not designed to test for these differences and we had no reasons to expect there to be any based on the existing literature. |
| Population characteristics | See above. |
| Recruitment | Participants were recruited by flyers and word-of-mouth at Columbia University. Our study is therefore limited to healthy younger adults, which may limit the generalizability of the results reported here. |
| Ethics oversight | All participants gave written, informed consent in accordance with the Institutional Review Board at Columbia University. |

Note that full information on the approval of the study protocol must also be provided in the manuscript.

# Field-specific reporting

Please select the one below that is the best fit for your research. If you are not sure, read the appropriate sections before making your selection.

☐ Life sciences ☒ Behavioural & social sciences ☐ Ecological, evolutionary & environmental sciences

For a reference copy of the document with all sections, see nature.com/documents/nr-reporting-summary-flat.pdf

# Behavioural & social sciences study design

All studies must disclose on these points even when the disclosure is negative.

| | |
|---|---|
| Study description | This was a quantitative experimental study. |
| Research sample | Thirty-five healthy younger adults from the Columbia University community participated in the experiment. Applying exclusions (see below) resulted in a final sample of 32 participants (21 female, 19-35 years old, mean = 24.17, sd = 4.11, 13-29 years of education, mean = 16.85, sd = 3.76). |
| Sampling strategy | The participants were based on a convenience sample from the Columbia University community. There was no power analysis performed, although our final sample size of 32 is a typical sample size for fMRI studies. |
| Data collection | During the behavioral training session, data was collected using PsychoPy, an Oculus Rift and Unity. In the fMRI session, data was collected using a 3 Tesla Siemens Magnetom Prisma scanner and PsychoPy. During the MRI scanner, there was an additional researcher present. The researcher was not blind to the experimental conditions of the study. |
| Timing | Data collection took place between December 2019 and March 2022. |
| Data exclusions | Three participants were excluded for technical issues with data collection, excessive motion (10% of TRs across all runs of the experiment marked as motion outliers by fMRIprep output), and dizziness inside the MRI scanner. |
| Non-participation | One participant declined to participate because they did not want to generate a story for the behavioral training session. |
| Randomization | This was a within-subjects study. As such, all participants were exposed to all experimental groups. |

# Reporting for specific materials, systems and methods

We require information from authors about some types of materials, experimental systems and methods used in many studies. Here, indicate whether each material, system or method listed is relevant to your study. If you are not sure if a list item applies to your research, read the appropriate section before selecting a response.

## Materials & experimental systems

| n/a | Involved in the study |
|---|---|
| ☒ | ☐ Antibodies |
| ☒ | ☐ Eukaryotic cell lines |
| ☒ | ☐ Palaeontology and archaeology |
| ☒ | ☐ Animals and other organisms |
| ☒ | ☐ Clinical data |
| ☒ | ☐ Dual use research of concern |
| ☒ | ☐ Plants |

## Methods

| n/a | Involved in the study |
|---|---|
| ☒ | ☐ ChIP-seq |
| ☒ | ☐ Flow cytometry |
| ☐ | ☒ MRI-based neuroimaging |

# Plants

Seed stocks: NA

Novel plant genotypes: NA

Authentication: NA

# Magnetic resonance imaging

## Experimental design

Design type: Event-related design

Design specifications: Prediction Task: 2 runs, 64 trials (32 trials per run), 11-15 seconds per trial, 3-8s jittered ITI between trials
Localizer Task: 4 runs, 64 trials (16 trials per run), 13 seconds per trial, 3-8s jittered ITI between trials

Behavioral performance measures: We recorded accuracy (correct button press) and response time. To ensure participants were performing the task, we compared accuracy to chance performance of 50% using a one-sample t-test ($t(31) = 61.08$, $p < 0.00001$).

## Acquisition

Imaging type(s): Functional

Field strength: 3 Tesla

Sequence & imaging parameters: Whole-brain data were acquired on a 3 Tesla Siemens Magnetom Prisma scanner equipped with a 64-channel head coil at Columbia University. Whole-brain, high-resolution (1.0 mm iso) T1 structural scans were acquired with a magnetization-prepared rapid acquisition gradient-echo sequence (MPRAGE) at the beginning of the scan session. Functional measurements were collected using a multiband echo-planar imaging (EPI) sequence (repetition time = 1.5s, echo time = 30ms, in-plane acceleration factor = 2, multiband acceleration factor = 3, voxel size = 2mm iso). Sixty-nine oblique axial slices were obtained in an interleaved order. All slices were tilted approximately -20 degrees relative to the AC-PC line. There were ten functional runs in total: two runs of the Anticipation Task, four runs of an Integration Task (not analyzed here), and four runs of the Localizer Task. Field maps were collected after the final functional scan to aid registration (TR = 679 ms, TE = 4.92 ms/7.38 ms, flip angle = 60°, 69 slices, 2 mm isotropic).

Area of acquisition: Whole-brain scan

Diffusion MRI: ☐ Used ☒ Not used

## Preprocessing

Preprocessing software: Results included in this manuscript come from preprocessing performed using fMRIPrep 1.5.2 (Esteban, Markiewicz, et al. (2018)65; Esteban, Blair, et al. (2018)66; RRID:SCR_016216), which is based on Nipype 1.3.1 (Gorgolewski et al. (2011)67; Gorgolewski et al. (2018)66; RRID:SCR_002502).

Normalization: Volume-based spatial normalization to one standard space (MNI152NLin2009cAsym) was performed through nonlinear registration with antsRegistration (ANTs 2.2.0), using brain-extracted versions of both T1w reference and the T1w template.

| | |
|---|---|
| Normalization template | The following template was selected for spatial normalization: ICBM 152 Nonlinear Asymmetrical template version 2009c [Fonov et al. (2009)73, RRID:SCR_008796; TemplateFlow ID: MNI152NLin2009cAsym]. |
| Noise and artifact removal | We conducted GLMs predicting whole-brain univariate BOLD activity from task and nuisance regressors (translation and rotation along the X, Y, and Z axes and their derivatives, motion outliers as determined by fMRIprep, CSF, white matter, framewise displacement, and discrete cosine-basis regressors for periods up to 125 seconds). |
| Volume censoring | NA |

## Statistical modeling & inference

| | |
|---|---|
| Model type and settings | We conducted multivoxel pattern analyses. To assess evidence for multivoxel representations of temporal structure, we obtained the correlation between (1) a given participant's (e.g., P1) cue screen activity pattern for each trial type (a given environment cued on a given path) in the Anticipation Task and (2) the remaining participants' (e.g., P2-P32) averaged patterns of activity for each of the environment templates from the corresponding map. We ordered these correlations as a function of distance to the cued environment on the order of the cued path and the uncued path. |
| Effect(s) tested | We tested whether the brain represented environments as a function of steps from the cue into the future and past on the cued vs uncued path. We conducted Gaussian modelling to test for such representations (see below). |

Specify type of analysis: ☐ Whole brain ☐ ROI-based ☒ Both

| | |
|---|---|
| Anatomical location(s) | Three a priori regions of interest (ROIs) were defined using environment-reliable voxels (see Manuscript) within anatomical or functional areas of interest. The V1-4 ROI was obtained from the probabilistic human visual cortex atlas provided in Wang et al.84 (threshold: $p = 0.50$). The hippocampus and insular cortex ROIs were both defined from the Harvard-Oxford probabilistic atlas in FSL (threshold: $p = 0.50$). We resampled the three ROIs onto the same MNI grid as the functional data (MNI152NLin2009cAsym), and then intersected them with our map of environment-reliable voxels ($r > 0.1$, see description above) to create conjunction ROIs in visual cortex, hippocampus, and insula. We also conducted a whole-brain searchlight analysis with custom Python code to test whether brain regions beyond our ROIs represented temporal structure in the hypothesized asymmetrical Guassian format. We looked for significant Gaussian representations in cubes with a side length of 7 voxels, moved throughout the whole brain volume with a step size of 2 voxels. We included only environment-reliable voxels within each cube, and only proceeded with the analysis of a cube if it contained at least 64 environment-reliable voxels. |
| Statistic type for inference (See Eklund et al. 2016) | NA |
| Correction | For our searchlight analyses, we corrected for multiple comparisons using the family-wise error rate correction ($p < 0.05$). |

## Models & analysis

| n/a | Involved in the study |
|---|---|
| ☐ | ☐ Functional and/or effective connectivity |
| ☐ | ☐ Graph analysis |
| ☐ | ☒ Multivariate modeling or predictive analysis |

| | |
|---|---|
| Functional and/or effective connectivity | *Report the measures of dependence used and the model details (e.g. Pearson correlation, partial correlation, mutual information).* |
| Graph analysis | *Report the dependent variable and connectivity measure, specifying weighted graph or binarized graph, subject- or group-level, and the global and/or node summaries used (e.g. clustering coefficient, efficiency, etc.).* |
| Multivariate modeling and predictive analysis | We obtained the correlation between (1) a given participant's (e.g., P1) cue screen activity pattern for each trial type (a given environment cued on a given path) in the Anticipation Task and (2) the remaining participants' (e.g., P2-P32) averaged patterns of activity for each of the environment templates from the corresponding map. We then fit an asymmetrical Gaussian curve to the resulting pattern similarity values arranged on the order of the cued or uncued path. To test whether the parameters of the Gaussian curve were consistent across participants, we fit the asymmetrical Gaussian curve on all but one participant's data (e.g. P2-32) and then measured the sum of squared errors (observed vs. predicted pattern similarity values) when using this curve to predict the held-out participant's data (e.g. P1). We repeated this procedure for each choice of held-out participant to obtain an average error value. We obtained a p-value by calculating the fraction of 10,000 shuffles that produced Gaussian fits with lower error than the fit for the correctly ordered data. We also calculated R2 as 1 minus the sum of squared errors of the Gaussian fit for the correctly ordered data divided by the mean sum of squared errors of the Gaussian fits for the shuffled data. |
